# Complete Procedure for Fabrication of a Fused Silica Ultrarapid Microfluidic Mixer Used in Biophysical Measurements

**DOI:** 10.3390/mi8010016

**Published:** 2017-01-07

**Authors:** Dena Izadi, Trieu Nguyen, Lisa J. Lapidus

**Affiliations:** 1 Department of Physics and Astronomy, Michigan State University, East Lansing, MI 48824, USA; Izadiden@msu.edu (D.I.); ngtrieu@pa.msu.edu (T.N.); 2 Department of Biochemistry and Molecular Biology, Michigan State University, East Lansing, MI 48824, USA

**Keywords:** microfluidic mixer, protein folding, KMPR, fused silica

## Abstract

In this paper we present a method to fabricate a fused silica microfluidic device by employing low viscosity KMPR photoresists. The resulting device is a continuous-flow microfluidic mixer based on hydrodynamic focusing. The advantages of this new fabrication method compared to the traditional approach using a poly-silicon mask are simplification, and time and cost reduction, while still preserving the quality and the performance of the mixers. This process results in devices in which the focusing channel has an aspect ratio of 10:1. The newly-fabricated mixer is successfully used to observe the folding of the Pin1 WW domain at the microsecond time scale.

## 1. Introduction

A microfluidic mixer based on hydrodynamic focusing made from silicon was first introduced in 1996 by Brody [1]. Since then, the mixer has been optimized and used intensively in the field of protein folding due to its extremely low mixing time, which is on the order of microseconds [2,3,4,5,6,7,8,9,10,11]. In contrast, conventional stopped-flow mixers have “dead times” of 1–5 ms, largely limited by turbulence. The device described in this work operates in the laminar flow regime such that channel dimensions are shrunk to the micron scale to allow linear flow rates of ~1–10 m/s without turbulence. Mixing is achieved by diffusion over short distances between different fluid streams. The fluid requirements of this device are also very low compared to turbulent devices, with protein consumption on the order of nL/s. The design described in this work has a mixing time of 8 μs (as defined as an 80% decrease in concentration [7]), one of the fastest reported to date, and has been measured experimentally and confirmed by simulation of the device by finite element analysis [3,4,12,13].

Since protein folding in a microfluidic mixer requires optical observation of the sample inside the mixing and downstream regions, these devices have been fabricated from transparent materials, such as borosilicate glass and fused silica. Previously [8], we reported a fabrication method for a fused silica microfluidic mixer utilizing a polycrystalline-silicon (poly-Si) mask. A thin layer of poly-Si is deposited on the fused silica surface in a low-pressure chemical vapor deposition (LPCVD) furnace. The mask pattern is then imprinted on the poly-Si surface by photolithography and etched into the poly-Si by deep reactive ion etching (DRIE). The poly-Si then functions as a mask during the fused silica etching step. Once the fused silica is etched, the poly-Si is removed via a xenon-difluoride etcher. This procedure requires highly specialized cleanroom machines for deposition, etching, and removal of the poly-Si mask and, consequently, is expensive and time consuming. Additionally it is not feasible to achieve a poly-Si layer of more than about 3 μm thickness due to stress on the wafer. As a result, the deepest channels achieved were 38 μm [14]. In order to overcome these disadvantages, in this work we report a new method to produce microfluidic mixers by using KMPR photoresist instead of poly-Si. It was recently reported that high viscosity KMPR photoresist (KMPR 1025) can be used to etch fused silica [15,16] but no completed devices have been made. Our new fabrication method for a complete microfluidic mixer uses low viscosity KMPR (KMPR 1005 and KMPR 1010), which has not been previously tried for fused silica fabrication. The protocol is less time consuming, lower cost, compared to the poly-Si deposition approach, and is able to etch deeper than 40 microns. The resulting channels in the mixers are of high quality with an aspect ratio as high as 10:1. The unetched surfaces remain atomically smooth, suitable for fusion bonding. These advantages make this fabrication method affordable and simple enough for use by researchers in the field of protein folding, in particular, and in the biotechnology community, in general.

## 2. Fabrication and Experiments

The fabrication of these microfluidic mixers was done in Robert H. Lurie Nanofabrication Facility at the University of Michigan and the Keck Microfabrication Facility at Michigan State University.

### 2.1. Chemicals and Materials

KMPR 1005 and KMPR 1010 were obtained from MicroChem Corp. (Westborough, MA, USA). Four-inch (0.525 and 0.170 mm thick) fused silica wafers were purchased from Plan Optik Company (Elsoff, Germany). In this paper, we mainly focus on using KMPR 1005 for the fabrication of the hydrodynamic focusing mixer (T-mixer) which should eventually have the channel depth of ~10 μm. For the demonstration of deep etching we use KMPR 1010, the results are shown in the supplementary material.

### 2.2. Process Flow

**Figure 1:**
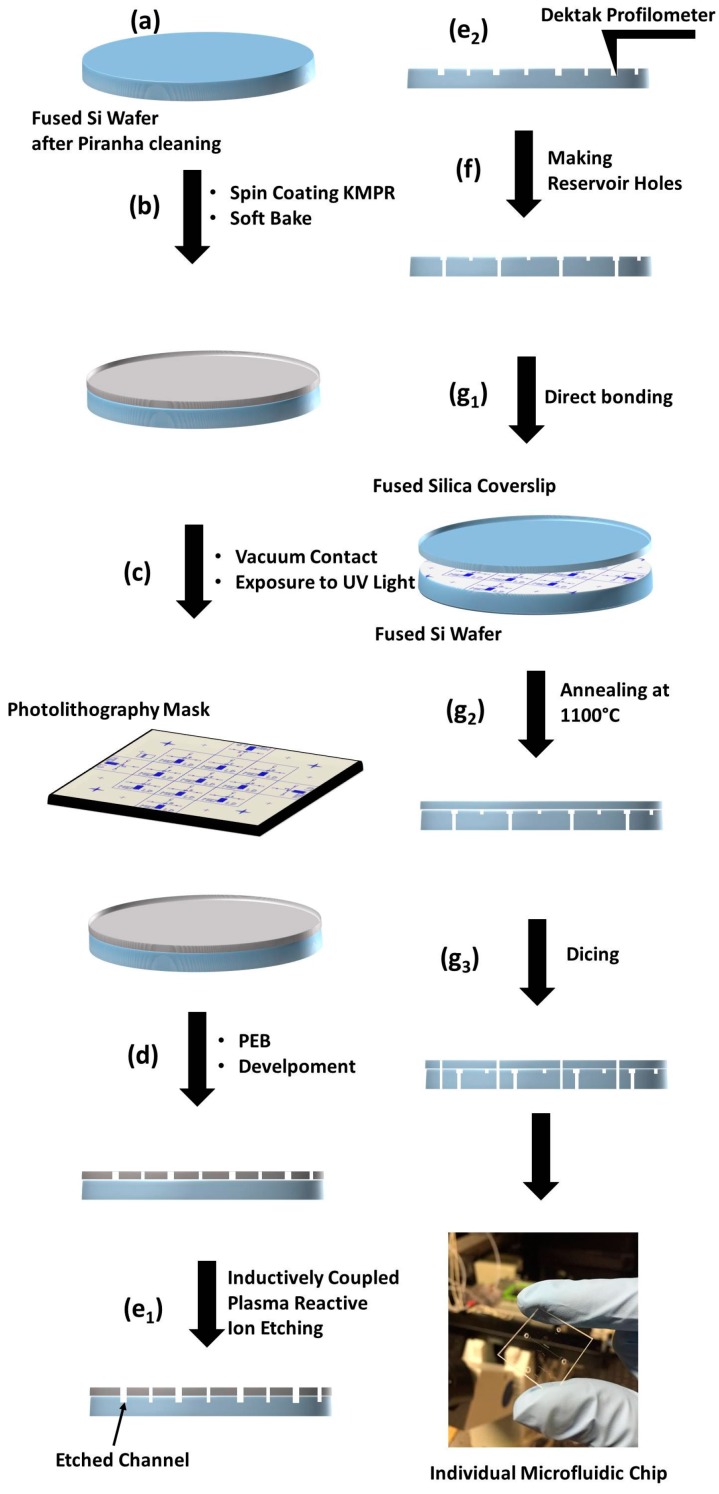
Schematic diagram of the procedure employed in the fabrication of T-mixer microfluidic chips.

The chip consists of two fused silica parts permanently bonded together. The top wafer (A) has a thickness of 170 micron. The bottom wafer (B) has a thickness of 525 micron. The following process (except step (g)) is for wafer B, (see Figure 1).

(a) Cleaning

The fused silica wafers were cleaned with Nano-strip, a stabilized formulation of sulfuric acid and hydrogen peroxide (cyantek.com), for 10 min at 60 °C, and then rinsed in de-ionized water over four cycles in a DI Quick Dump Rinse tank for 5 min. The wafer was then dried with nitrogen gas.

(b) Photoresist coating

KMPR was spin-coated on the wafer using an ACS200 cluster tool (SUSS MicroTec, Munich, Germany). The viscosity of KMPR 1005 is 95 centistokes (cSt). A soft-bake at 100 °C for 7 min was performed by the ACS200 cluster tool before dispensing for 3 s at 4500 rpm.

(c) Photolithography

A negative photolithography mask was fabricated from Compugraphics USA (Fremont, CA, USA). A Karl Suss MA-6/BA-6 (SUSS MicroTec, Garching, Germany) contact aligner was used to expose the mask and substrate to UV light. This aligner uses a wedge error compensation (WEC) head to apply 1.0 bar pressure for high contact between the mask and the wafer. The UV source emits 20 mW/cm^2^ at 405 nm. The exposure time is 5.5 s. After exposure, the substrate is baked at 100 °C for 7 min. The image of the mask will be visible in the KMPR, confirming there was sufficient exposure.

(d) Developing

Development of the pattern uses AZ-300 MIF developer (EMD Performance Materials, Somerville, NJ, USA) for 135 s. After development, the sample was spray rinsed for about 60 s with de-ionized water and dried with nitrogen gas.

(e) Etching

The channels were etched using reactive ion etching optimized for oxide substrates. The wafer was mounted on a six-inch silicon wafer to provide mechanical support and etched with the SPTS APS Dielectric Etch tool (STS Glass Etcher, SPTS technologies, Newport, UK). The etch rate was 0.50 μm/min. Therefore, 10 μm deep channels required 20 min of etching. The remaining KMPR was removed with REMOVER PG (MicroChem Corp., Westborough, MA, USA), a proprietary *N*-methyl-2-pyrrolidone-based solvent. The wafers were immersed for 30–45 min at 65–80 °C and then left to soak in the solvent overnight. The wafers were rinsed with isopropyl alcohol and de-ionized water, and the channel depths were confirmed with a Dektak^®^ profilometer (Bruker Corporation, Billerica, MA, USA) measurement.

(f) Sandblasting

A high-precision sandblaster (Crystal Mark Inc., Glendale, CA, USA) was used to create the openings which allowed fluid flow into the channels from the sample reservoirs sealed by O-rings to the back of the mixer. The aluminum powder was comprised of 27.5 micron diameter particles.

(g) Bonding and dicing

Prior to bonding, both wafers A and B are cleaned with RCA reverse cleaning for a total of 100 min. Then the two wafers are placed together and a pressure from a single finger will cause the pre-bonding front to expand out to the perimeter. The wafer is then baked at 1100 °C for 2 h and the individual chips diced using a laser engraver (VLS2.30, Universal Laser Systems, Scottsdale, AZ, USA).

### 2.3. Protein Refolding Experiments

The resulting microfluidic mixers are used to investigate the folding of the Pin1 WW domain by monitoring of Trp fluorescence under a special scanning confocal microscope. The experimental setup is the same as that shown in the previous work [8]. The protein is first unfolded by dissolving lyophilized protein (300 μM) in 6 M guanidine hydrochloride (GnHCl). Folding was initiated by mixing them with 100 mM potassium phosphate buffer (PPB, pH 7.0) in the microfluidic mixer. Mixing is achieved by flowing the buffer at the side channels at ~100 times the flow rate of the unfolded protein in the center channel, constricting the protein stream to a jet ~100 nm wide [8]. After mixing, the flow proceeds down the exit channel at a constant rate. During the folding experiment, the Trp fluorescence decay along the jet was measured at different locations with a photon counter.

## 3. Results and Discussion

### 3.1. Microfluidic Mixers

**Figure 2:**
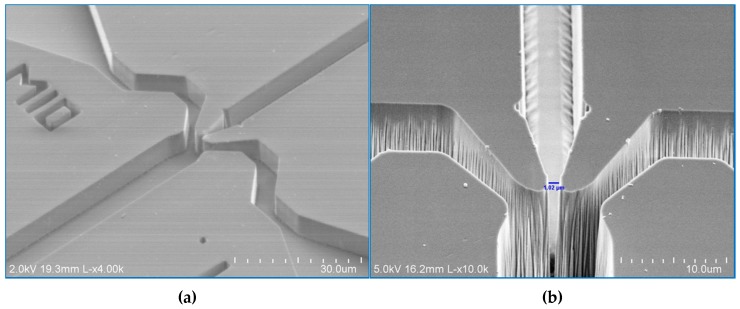
(**a**) SEM of a uniform KMPR 1005 layer after developing step; and (**b**) SEM of the etched fused silica.

Figure 2a shows the SEM of a uniform layer of KMPR 1005 after the developing step. The thickness of this layer is measured by using Dektak 8M Surface Profilometer and is ~7 μm. Figure 2b shows the mixing region after etching. At the end of the center channel, as shown in Figure 2b, the width of the focusing nozzle is ~1 μm (the blue line in Figure 2b). The depth of the channel, determined using Dektak, is ~10 μm. These measurements indicate that, by using KMPR 1005 photoresist, we obtain in the focusing region an aspect ratio of 10:1, which is comparable to previous work using a poly-Si mask [8], while requiring fewer fabrication steps, hence reducing the experimental time and cost.

**Figure 3:**
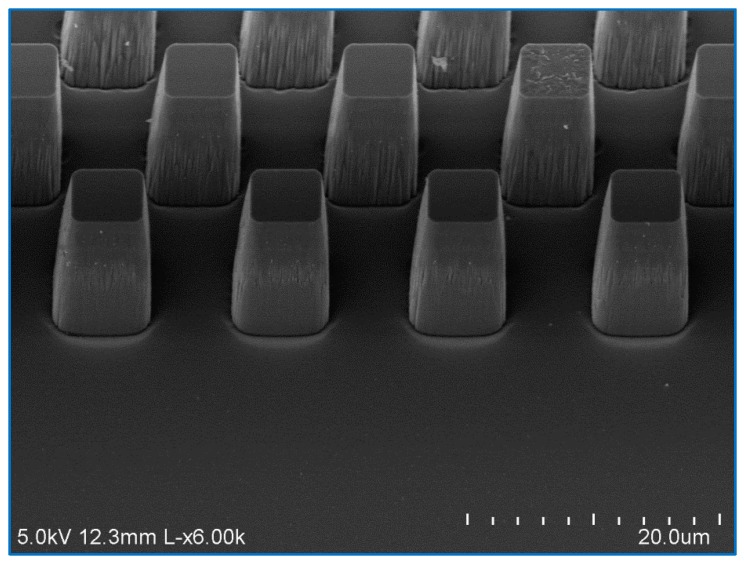
SEM of a filter region made of a series of fused silica micron-sized posts.

**Figure 4:**
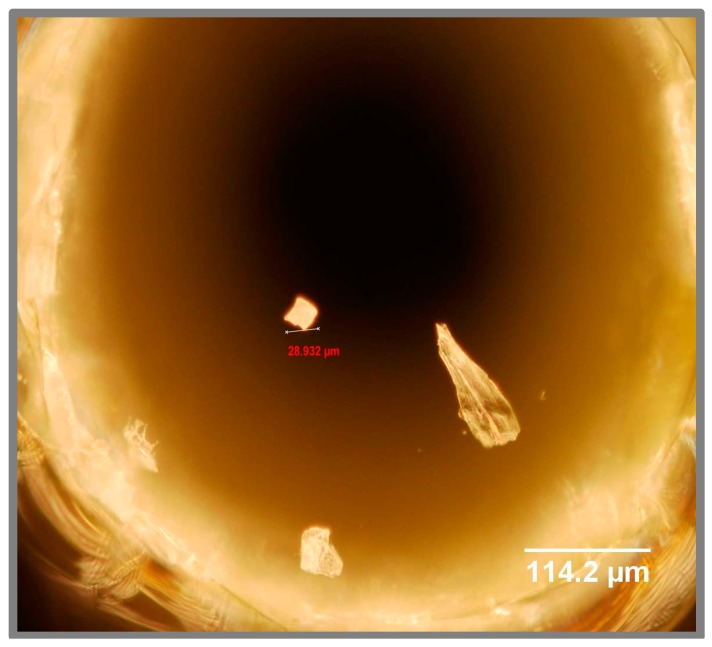
Microscope picture of the inlet hole made from a drilling machine with resulting shards falling off the fused silica surface.

**Figure 5:**
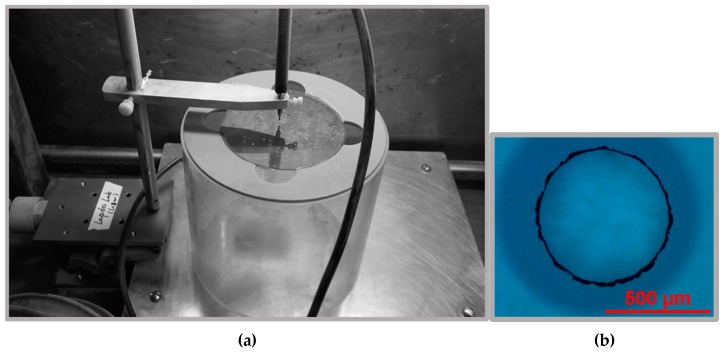
(**a**) Sandblaster set-up; and (**b**) microscope picture of the inlet hole made from the sandblaster.

The mixing time is determined, in part, by the width of the nozzles in the mixing region. This feature is limited to ~1 μm by the resolution of our lithography. However, a narrow nozzle also makes the center channel prone to clogging by particle contamination. The chip design features a filter region made of a series of fused silica micron-sized posts [17] to catch particulates within the flow streams (see Figure 3), and cleaning with Piranha solution can dissolve most organic particulates. However, this system does not catch one common type of particulate on the small, irregular shards of fused silica created during the drilling of inlet holes (Figure 4) where the fluid enters the channels. These shards tend to stick strongly to the inside of the hole and are difficult to remove by washing or air pressure prior to bonding. However, continuous flow during use of the chip will eventually dislodge such shards that may be caught in the mixing region, frequently disabling the device. We have, therefore, improved another step of the fabrication method, using a sandblaster to create the openings instead of a diamond-tipped drill. Sandblasting with 27.5 μm aluminum oxide powder does not leave shards of fused silica in the resulting holes (Figure 5) and, therefore, improves the reproducibility of fabricating these chips.

For the results of using of KMPR 1010, please refer to the supplementary material of this paper.

### 3.2. Protein Folding

**Figure 6:**
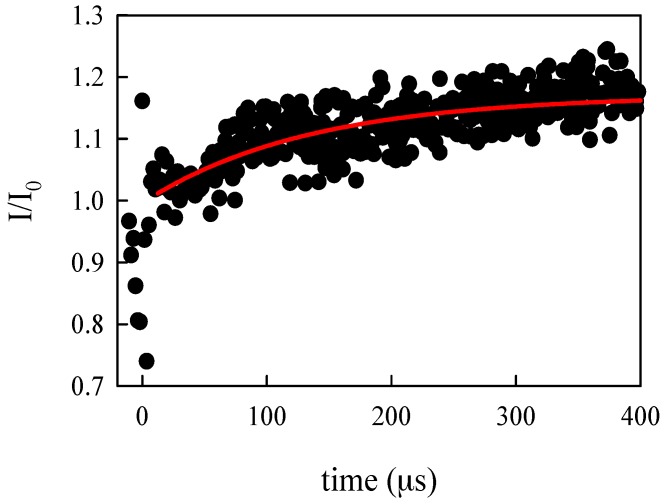
Relative fluorescence of the refolding experiment versus time.

We used this hydrodynamic focusing mixer and the scanning confocal setup to capture the fluorescence intensity of the folding of the Pin1 WW domain. Details of the optical instrument are discussed in [8]. Briefly, the fluorescence within the mixer is observed with a custom-built confocal microscope. The Trp fluorescence is excited by an argon-ion laser frequency doubled to emit 258 nm light. The light is focused to ~1 μm spot by a fused silica microscope objective and emitted fluorescence is captured by the same objective and detected by a photon counting module. The chip is scanned across the objective to measure the fluorescence at different positions in the supply and exit channel. The channel position is converted to time by the known flow rate. The fluorescence intensity during folding (I) was normalized by a baseline measurement (I_0_) where the protein is mixed with the same denaturant solution it was already dissolved in. This normalization removes optical artifacts due to surface inconsistencies from etching and the large drop in fluorescence during formation of the jet. Figure 6 shows the relative fluorescence vs. time. Data collected in the center channel above the mixing region is assigned a negative time. Any change in fluorescence during the mixing time would suggest extra phases that cannot be resolved by this device. For this protein, as can be seen in Figure 6, there does not appear to be a substantial “burst phase” (hydrophobic collapse). The rise in fluorescence is fit to a single exponential with a rise time of 133 μs (red curve in Figure 6), in agreement with the folding rate measured by laser T-jump [18].

## 4. Conclusions

We developed a new procedure for making an ultrafast fused silica microfluidic mixer by using low-viscosity KPMR photoresist (KMPR 1005 and KMPR 1010). The new procedure is simpler, less time consuming, and less expensive, without compromising quality or performance of the microfluidic device compared to the previous method using poly-Si as the mask. We successfully demonstrated the utilization of the newly-fabricated mixer to investigate the folding of the Pin1 WW domain at the microsecond time scale. The new procedure developed in this work can also be employed to other applications, such as lab-on-a-chip or biophysical measurements, which require the microfabrication of fused silica devices.

## Supplementary Materials

The following are available online at www.mdpi.com/2072-666X/8/1/16/s1, Figure S1: SEM image of KMPR 1010 layer after developing on the fused silica, Figure S2: SEM image of etched fused silica channels after removing KMPR1010, Table S1: Fabrication process for KMPR1010, Figure S3: the complete image of the structure of the ultrarapid mixer.
