# Supplementary material for: Complete Procedure for Fabrication of a Fused Silica Ultrarapid Microfluidic Mixer Used in Biophysical Measurements
Source: Micromachines (Basel). 2017 Jan 7;8(1):16. doi: 10.3390/mi8010016 (PMC6189705; doi:10.3390/mi8010016)
Supplement: Supplementary file 1 [file micromachines-08-00016-s001.pdf]

Supplementary Materials: Complete Procedure for Fabrication of a Fused Silica Ultrarapid Microfluidic Mixer Used in Biophysical Measurements

Dena Izadi, Trieu Nguyen and Lisa Lapidus

Deep Etching Fused Silica with KPMR 1010

Figure S1 shows the SEM image of uniform KMPR 1010 layer after developing. The thickness of this layer is ~17 μm measured using a Dektak 8M Surface Profilometer (Bruker Corporation, Billerica, MA, USA) (see Table S1). Figure S2 shows the SEM of the etched fused silica channel after removing KMPR 1010. The depth of this channel is ~45 μm as measured by the Dektak 8M Surface Profilometer.

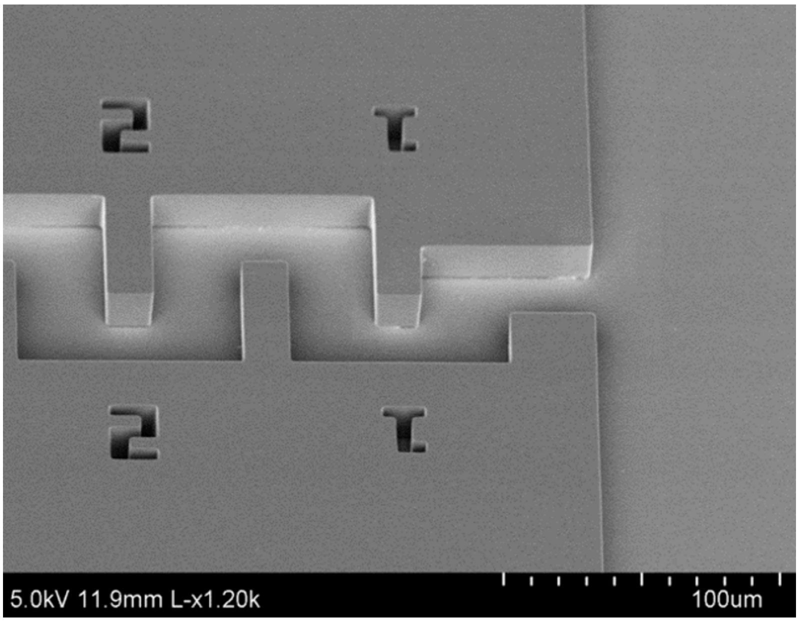

Figure S1. SEM image of KMPR 1010 layer after developing on the fused silica.

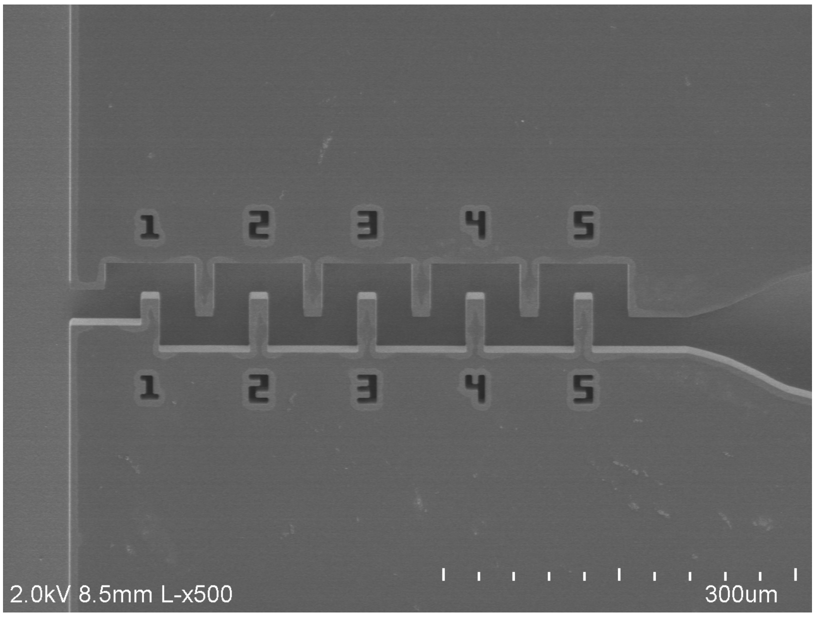

Figure S2. SEM image of etched fused silica channels after removing KMPR 1010.

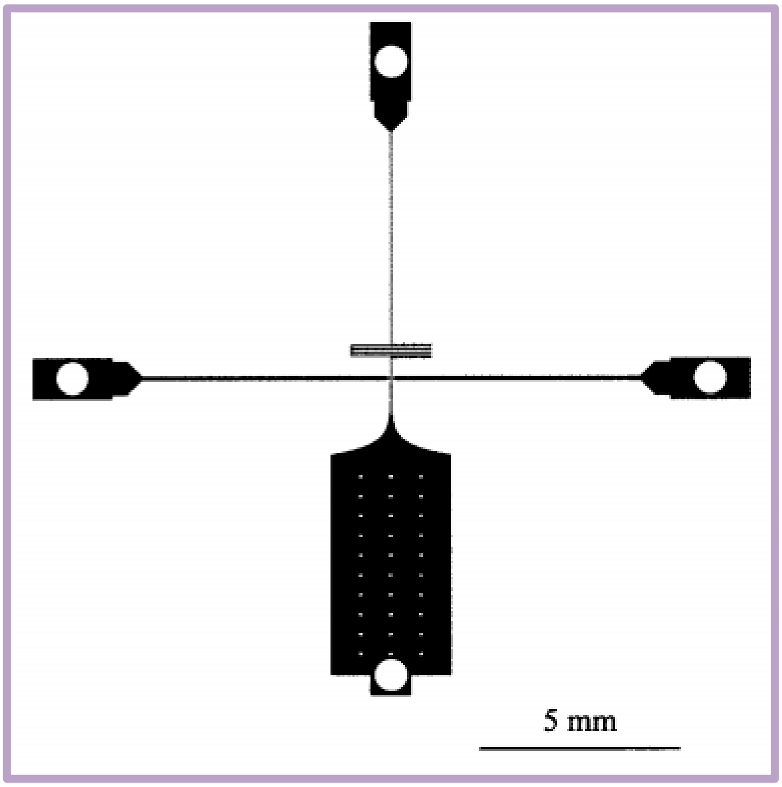

Figure S3. Layout of individual chip [1].

Table S1. Fabrication process for KMPR1010.

Process Parameter	Process Tool	Serpentine Mixer Design
Photoresist	ACS 200 Cluster	KMPR® 1010
Photoresist spin speed	ACS 200 Cluster	1500 RPM
Average resist Thickness	Dektak 8M Surface Profilometer	17.0 µm
UV exposure time	Karl Suss MA6	18.0 s
Development time	AZ 300 MIF	285 s
Total etch time	STS glass etcher	90 min
Etched channel depth	Dektak 8M	45.0 µm

Reference

1. Waldauer, S.A. Early Events in Protein Folding Investigated through Ultrarapid Microfluidic Mixing. Ph.D. Thesis, Michigan State University, East Lansing, MI, USA, 2010.

© 2017 by the authors. Submitted for possible open access publication under the terms and conditions of the Creative Commons Attribution (CC-BY) license (http://creativecommons.org/licenses/by/4.0/).
